# Supplementary figures and images for: Bayesian inference of COVID-19 spreading rates in South Africa
Source: PLoS One. 2020 Aug 5;15(8):e0237126. doi: 10.1371/journal.pone.0237126 (PMC7406053; doi:10.1371/journal.pone.0237126)

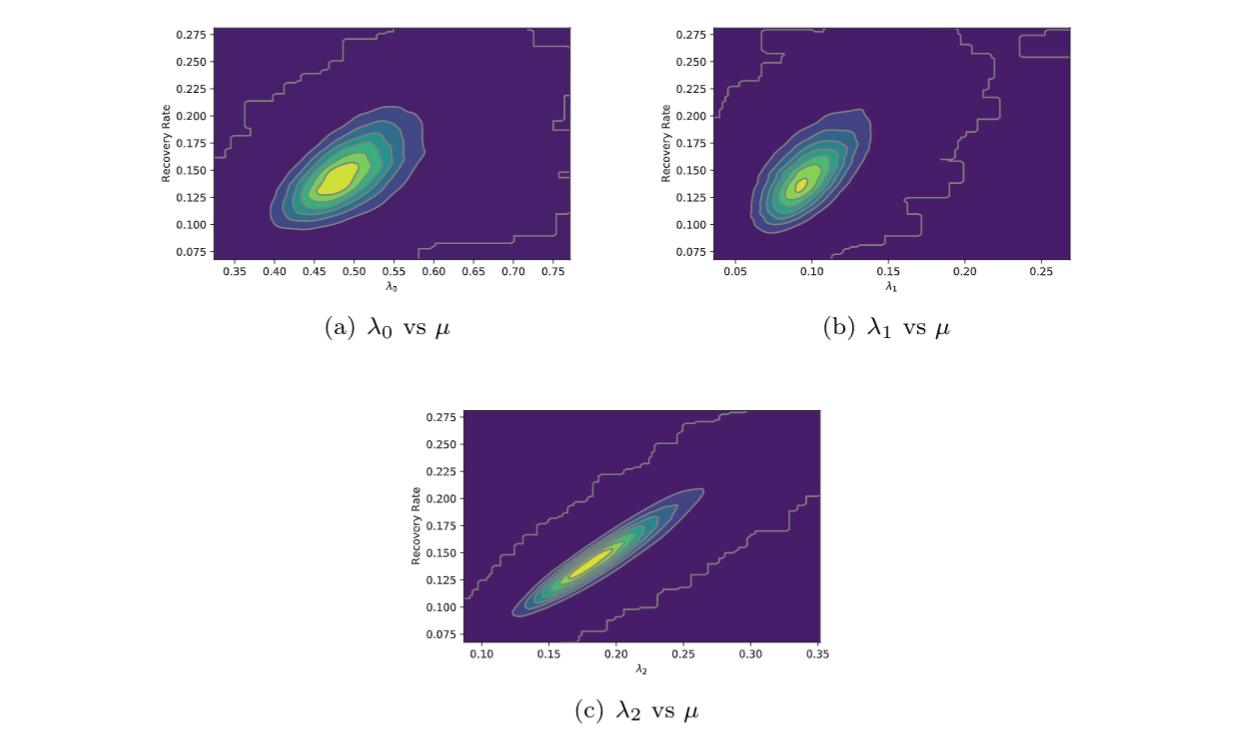

Supplement: S1 Fig — The high joint density areas (in yellow) indicate likely values of R0. The baseline mean R0 estimate in S1 Fig (a) is 3.278, the first change point estimate in Fig S1 Fig (b) is 0.655 while the second change point in S1 Fig (c) has resulted in a mean R0 estimate of 1.304. (TIFF) [file pone.0237126.s001.tiff]

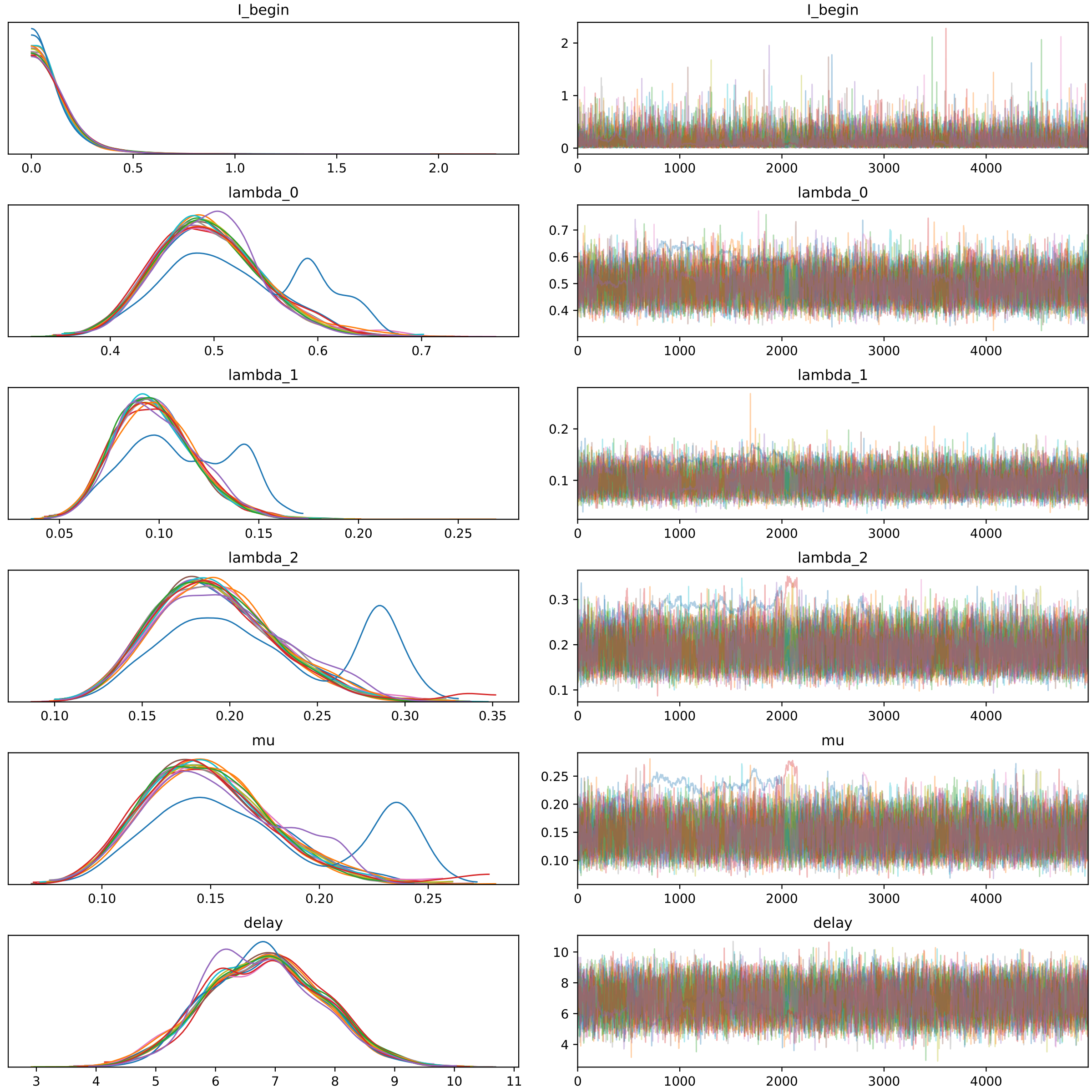

Supplement: S2 Fig — (TIFF) [file pone.0237126.s002.tiff]

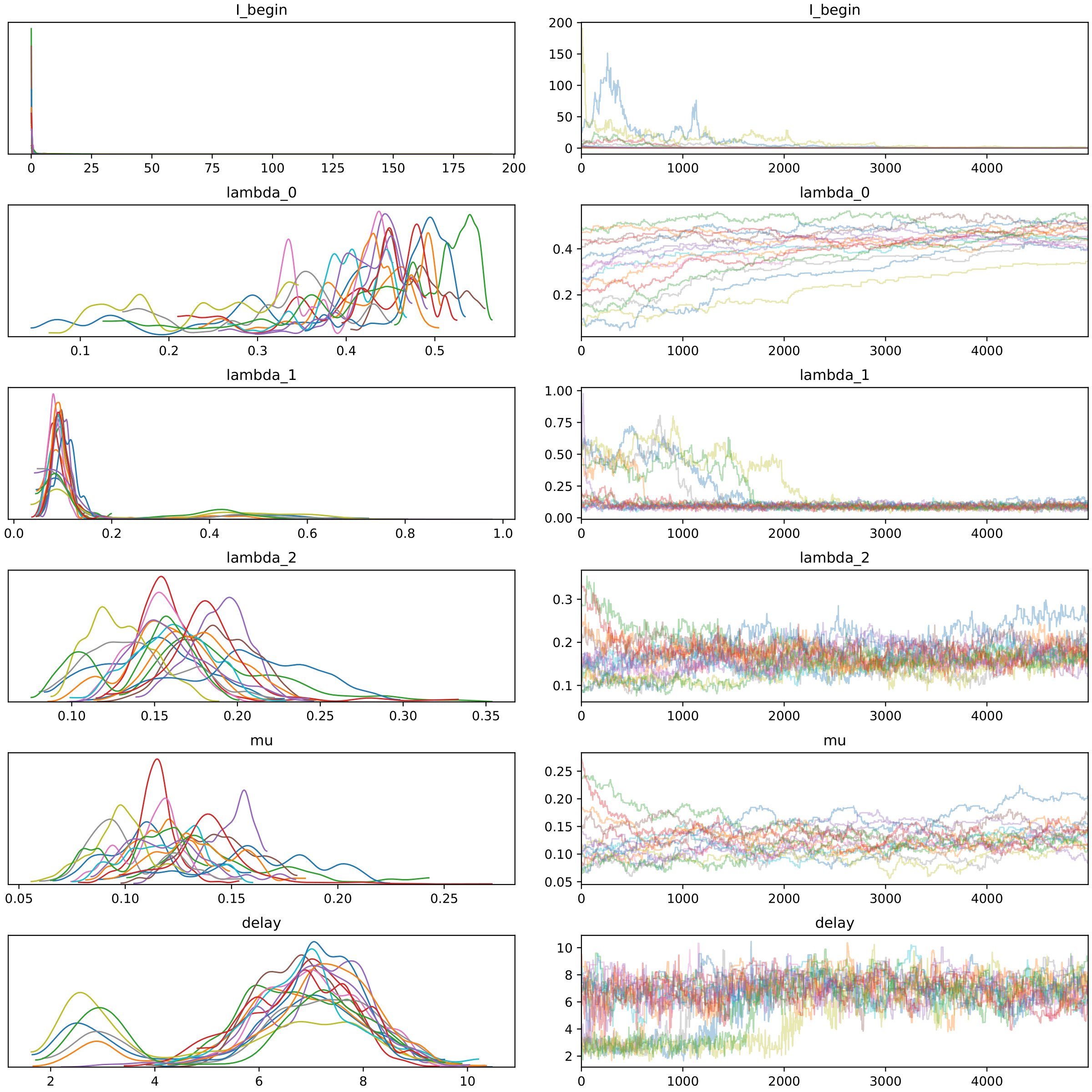

Supplement: S3 Fig — (TIFF) [file pone.0237126.s003.tiff]

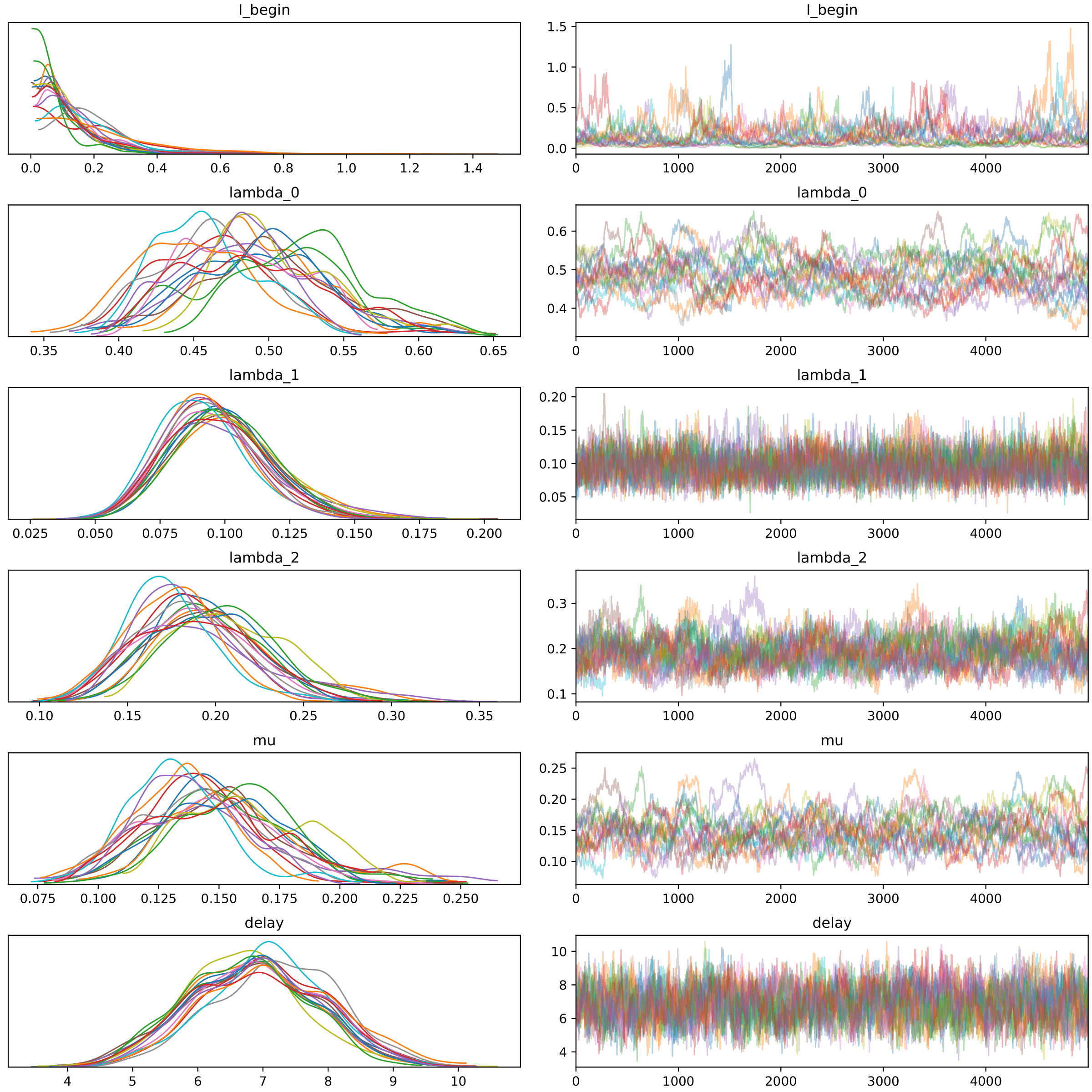

Supplement: S4 Fig — (TIFF) [file pone.0237126.s004.tiff]

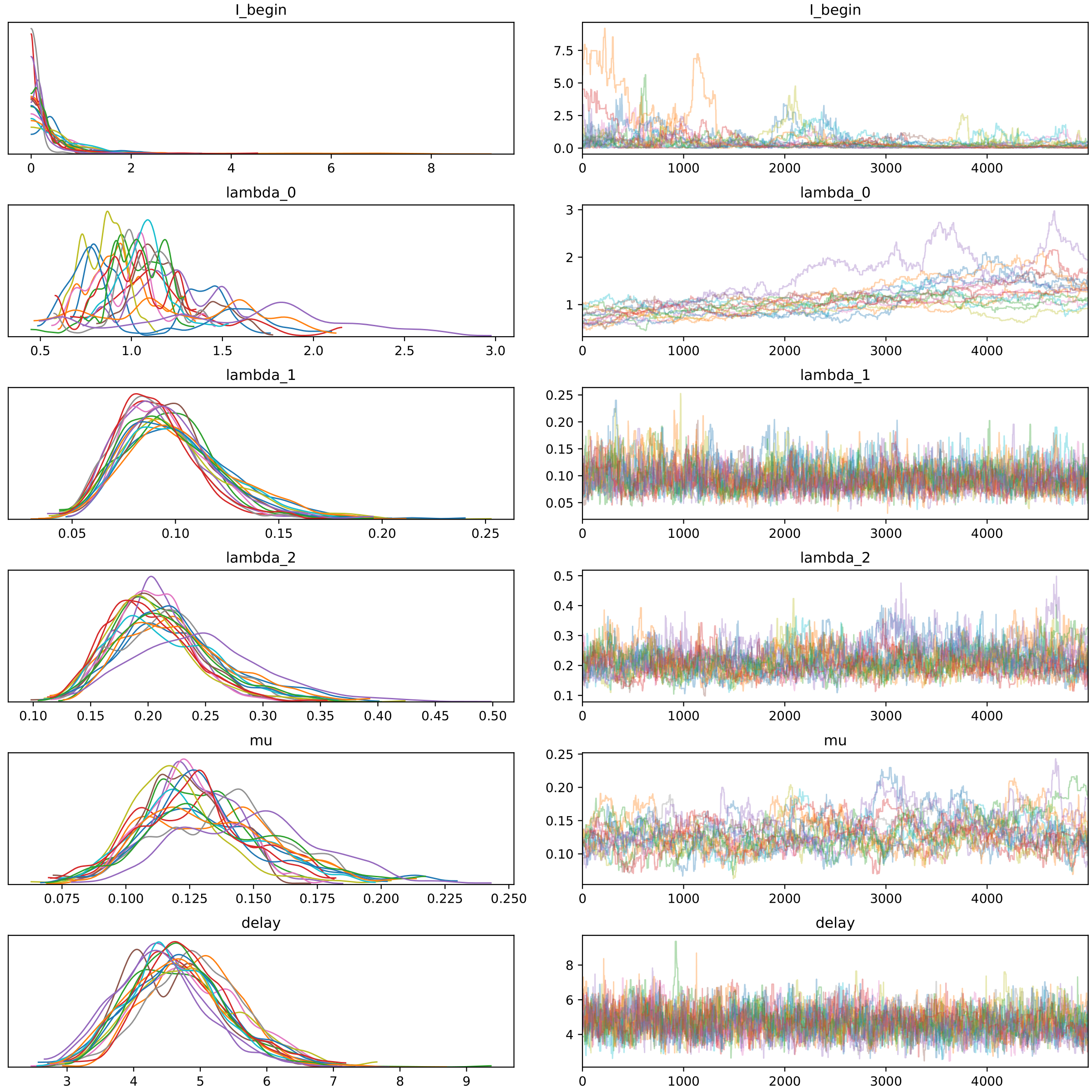

Supplement: S5 Fig — (TIFF) [file pone.0237126.s005.tiff]

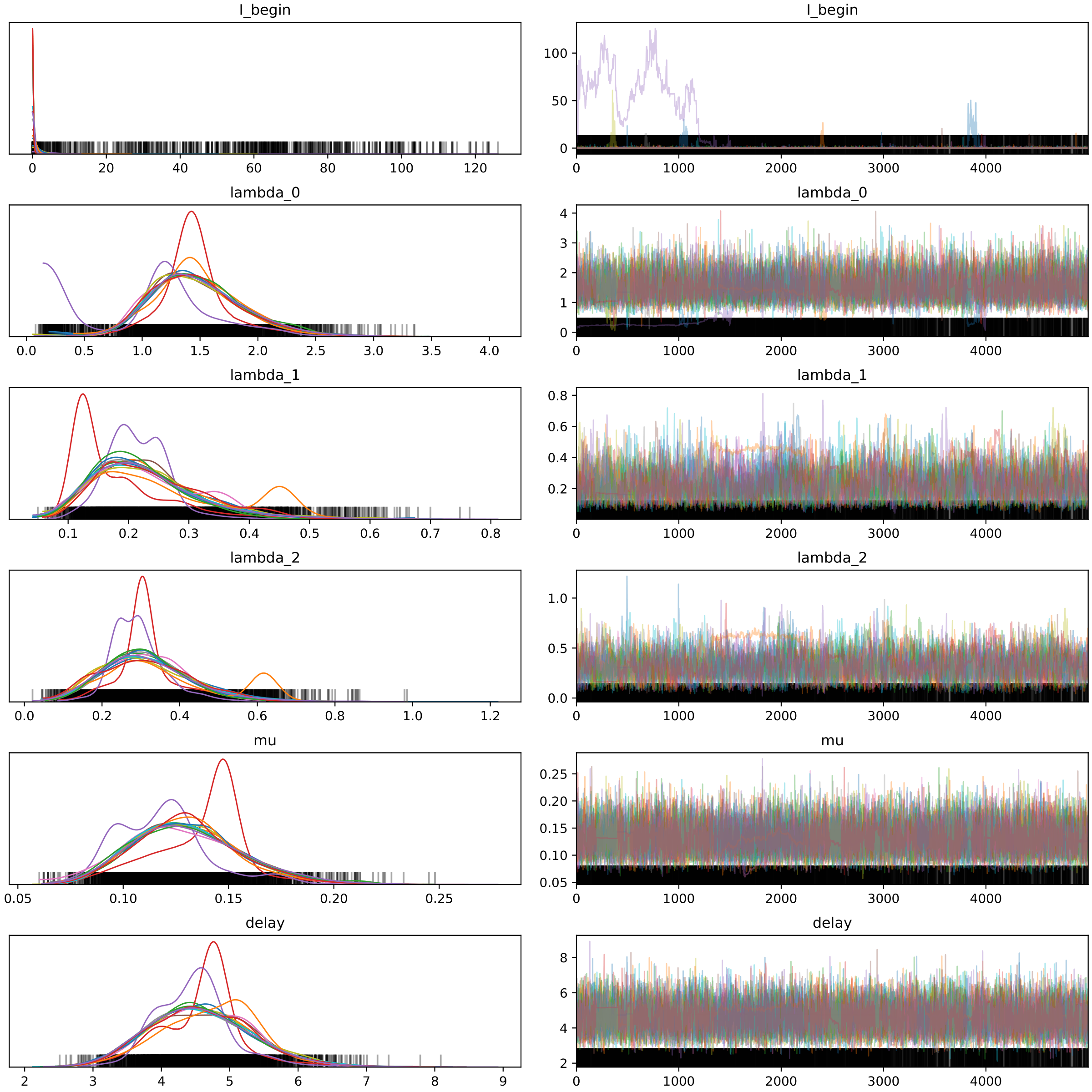

Supplement: S6 Fig — (TIFF) [file pone.0237126.s006.tiff]

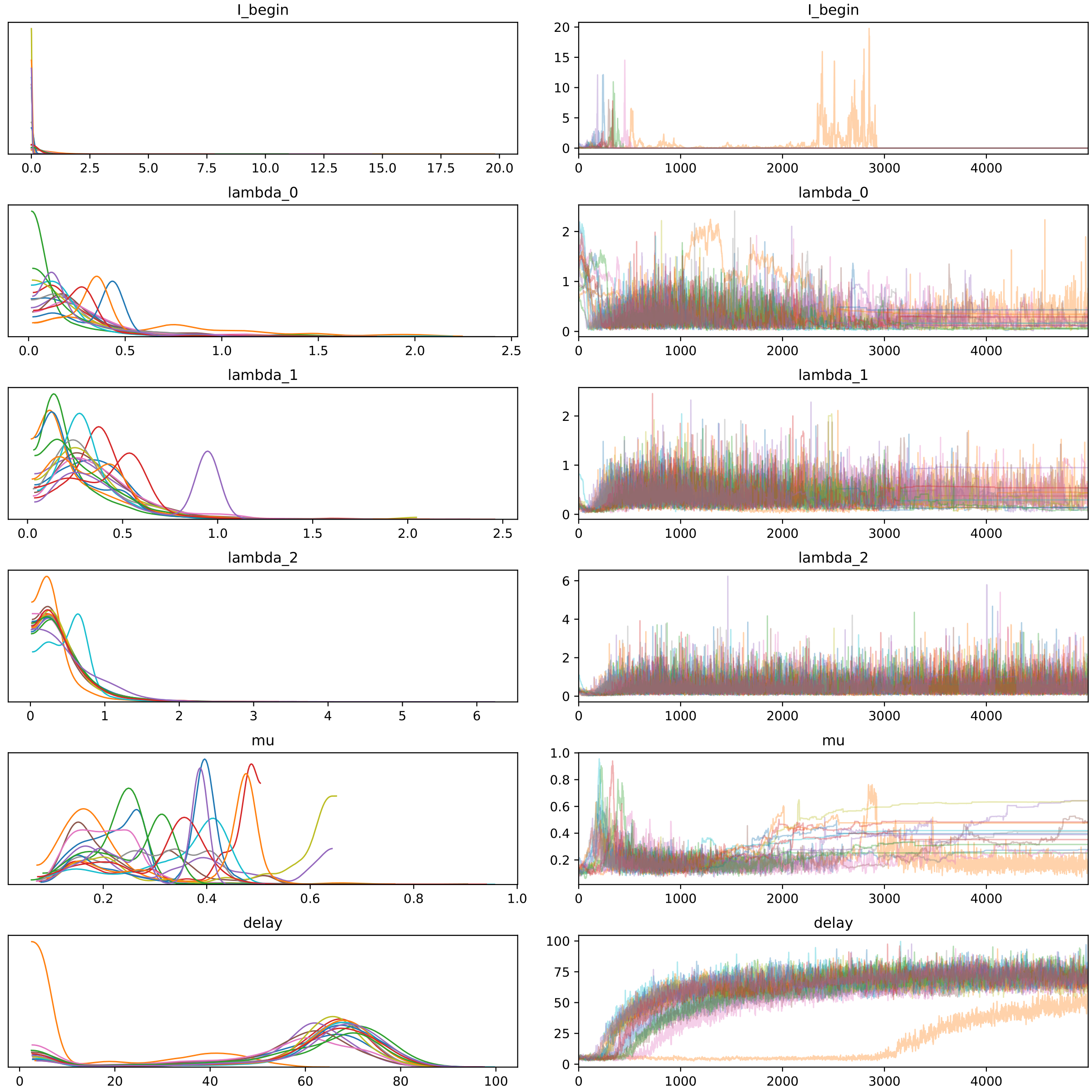

Supplement: S7 Fig — (TIFF) [file pone.0237126.s007.tiff]
