# Supplementary material for: Bayesian inference of COVID-19 spreading rates in South Africa
Source: PLoS One. 2020 Aug 5;15(8):e0237126. doi: 10.1371/journal.pone.0237126 (PMC7406053; doi:10.1371/journal.pone.0237126)
Supplement: Attachment — Submitted filename: Response to Reviewers.pdf [file pone.0237126.s008.pdf]

Dear Editor

Kindly find our responses to reviews below.

Best,
Rendani Mbuvha

Responses to Academic Editor

1. Please ensure that your manuscript meets PLOS ONE's style requirements, including those for file naming. The PLOS ONE style templates can be found at https://journals.plos.org/plosone/s/file?id=wjVg/PLOSOne_formatting_sample_main_body.pdf and https://journals.plos.org/plosone/s/file?id=ba62/PLOSOne_formatting_sample_title_authors_affiliations.pdf

Response

We have made changes in line with the formatting guidelines, including:

- *Changing the affiliation of one author from 'office of the vice-chancellor(his office)' to the institute that he is affiliated to at the university*
- *Changing 'figure' references to 'fig'*
- *Moving supporting information to an appropriately named appendix*
- *Changing subsection headings accordingly*

2.We noticed you have some minor occurrence of overlapping text with the following previous publication(s), which needs to be addressed:
- arXiv:1906.06382v1
-https://www.medrxiv.org/content/10.1101/2020.04.07.20057133v2
In your revision ensure you cite all your sources (including your own works), and quote or rephrase any duplicated text outside the methods section. Further consideration is dependent on these concerns being addressed.

Response:

We have made changes in terms of rewriting, rephrasing(with referencing) and inserting quotations in areas where there was previous overlapping text with our earlier preprints outside the methods section. Please see highlighted changes.

Please also ensure you have described the source of your data in the methods section, including a link or citation which can be used to access the data.

Response:

We have added a description of our data source, including the necessary citation.

3. Please ensure that you refer to Figures 10,11 & 12 in your text as, if accepted, production will need this reference to link the reader to the figure.

Response:

These figures have now been moved to the supporting information files with the relevant references included in the main text.

4. Please upload a copy of Supporting Information Appendix S1 which you refer to in your text on page 11.

Response:

Now included as separate files - (this mainly consists of the previous 'figures' 10-12 and other diagnostic plots)

Responses to Reviewer 1

I recommend this paper for publication because of its timeliness and well described methods and would suggest a few minor revisions:

- In table 2 the authors list compare different sampling methods by their LOO statistic. As far as I understand it this is not useful and misleading. The LOO statistic was developed to compare different models (which are also correctly shown in this table). The lower LOO statistic of the MH sampling probably arises from a incomplete sampling of the posterior which could potentially be fixed letting the chains run longer and/or with a longer burn-in period. A useful statistic to check convergence is the Rhat statistic (https://docs.pymc.io/api/stats.html#pymc3.stats.rhat).

Response:

We agree with the reviewer. We have revised table 2 to only refer to the comparison between models rather than include samplers. We have also increased the number of chain runs to 5000 and increased burn-in to 1000 runs with ten concurrent chains rather than four to increase the likelihood of convergence.

- The scale of the lambda_2 prior in the table 1 doesn't seem to match the prior distribution plotted in Fig. 4. The authors could also think about whether the lambda 1 and 2 priors are well motivated, and eventually make them wider/more uninformative. The posterior of the Lambda 1 prior is in the tail of the prior distribution (Fig. 4).

Response:

We have now changed both priors for lambda 1 and lambda 2 to a wider and relatively less informative LogNormal(log(0.4),0.7).

The change in priors and running the chains for longer marginally changes mean estimates of posterior parameters -- this does not seem to change the overall findings in a material way.

- In the algorithm of the HMC, in the third line of the leapfrog loop, the closing bracket after "p(t" should be removed.

Response:

We have amended the formula typo accordingly.
